# Perspective Strategies for Interventions in Parkinsonism: Remedying the Neglected Role of TPPP

**DOI:** 10.3390/cells13040338

**Published:** 2024-02-14

**Authors:** Judit Oláh, Vic Norris, Attila Lehotzky, Judit Ovádi

**Affiliations:** 1 Institute of Molecular Life Sciences, HUN-REN Research Centre for Natural Sciences, H-1117 Budapest, Hungary; lehotzky.attila@ttk.hu (A.L.); ovadi.judit@ttk.hu (J.O.); 2 Laboratory of Bacterial Communication and Anti-Infection Strategies, EA 4312, University of Rouen, 76821 Mont Saint Aignan, France; victor.norris@univ-rouen.fr

**Keywords:** Parkinsonism, methodologies, alpha-synuclein, TPPP, drug targeting

## Abstract

Neurological disorders such as Parkinsonism cause serious socio-economic problems as there are, at present, only therapies that treat their symptoms. The well-established hallmark alpha-synuclein (SYN) is enriched in the inclusion bodies characteristic of Parkinsonism. We discovered a prominent partner of SYN, termed Tubulin Polymerization Promoting Protein (TPPP), which has important physiological and pathological activities such as the regulation of the microtubule network and the promotion of SYN aggregation. The role of TPPP in Parkinsonism is often neglected in research, which we here attempt to remedy. In the normal brain, SYN and TPPP are expressed endogenously in neurons and oligodendrocytes, respectively, whilst, at an early stage of Parkinsonism, soluble hetero-associations of these proteins are found in both cell types. The cell-to-cell transmission of these proteins, which is central to disease progression, provides a unique situation for specific drug targeting. Different strategies for intervention and for the discovery of biomarkers include (i) interface targeting of the SYN-TPPP hetero-complex; (ii) proteolytic degradation of SYN and/or TPPP using the PROTAC technology; and (iii) depletion of the proteins by miRNA technology. We also discuss the potential roles of SYN and TPPP in the phenotype stabilization of neurons and oligodendrocytes.

## 1. Introduction

There are millions of people suffering from Parkinsonism in the world and there is neither a medicine to cure the disease nor an appropriate test to detect the illness at an early stage and to follow the disease/treatment processes at the clinical level [1,2]. Alpha-synuclein (SYN) plays a key role in Parkinsonism, which includes Parkinson’s disease (PD), dementia with Lewy bodies (DLB), and multiple system atrophy (MSA) [3,4,5,6,7]. Parkinsonism can be characterized by the classical motor symptoms (such as resting tremor, rigidity, bradykinesia, and freezing of gait) as well as non-motor features (including constipation, depression, sleep disorder, cognitive impairment, and dementia) [6]. In the case of MSA, patients may exhibit either Parkinsonism (MSA-P) or cerebellar ataxia (MSA-C). Despite the many shared symptoms, these diseases differ in the rate of disease progression. Median survival is much shorter for DLB and MSA patients than for PD ones. Current treatments include different therapies (mostly affecting dopamine metabolism, for example, by levodopa), deep brain stimulation, active and passive immunotherapy, and other options for transformative treatment (such as stem cell transplants or gene-targeted treatments) [1,8,9]. Levodopa relieves the motor symptoms through the replacement of lost dopamine; however, MSA-P patients are usually less responsive to levodopa therapy, and it can also worsen neuropsychiatric symptoms [6].

A major characteristic of these neurological disorders is the atypical protein assembly (proteopathy) leading to cell death, which is one of the key mechanisms of many neurodegenerative diseases. In pathological conditions, the aggregation of SYN results in the formation of insoluble fibrils, which had long been considered the primary structural components of synucleinopathies [3,4,10]. However, there are also striking differences between the different synucleinopathies as there are differences in the brain regions affected and in the cell types with inclusion bodies [6]. SYN aggregation is typically observed in the substantia nigra pars compacta in PD and in the cerebral cortex and hippocampus in DLB, while the olivopontocerebellar, nigrostriatal, and autonomic systems are affected in MSA. In PD and DLB, SYN-bearing Lewy bodies and Lewy neurites can be observed in neurons, while MSA is characterized by aggregated SYN in glial cytoplasmic inclusions [3,4,5]. The pathological SYN strains from Lewy bodies and glial cytoplasmic inclusions seem to be conformationally and biologically distinct [11]. 

In the last decade, however, Tubulin Polymerization Promoting Protein (TPPP) has also emerged as one of the principal actors in the processes underlying neurological disorders [12,13,14]. Pathological interactions of TPPP with SYN can induce SYN assembly that leads to the formation of inclusions as pathological hallmarks characteristic of PD, DLB, and MSA [12,15]. TPPP, as a Neomorphic Moonlighting Protein, plays an important role in both physiological and pathological conditions without alterations at the gene level [16]. TPPP is expressed specifically in oligodendrocytes (OLGs) in the normal brain during differentiation of the progenitor cells, where it is crucial to the formation of projections that results in the ensheathment of the axons [17]. This ensheathment enables highly efficient signal transmission; moreover, OLGs also provide metabolic and trophic support to the neurons [18]. TPPP is not present in astroglia or microglia [17,19]. In physiological conditions, TPPP modulates the dynamics and stability of the cytoskeletal microtubule system via its bundling and tubulin acetylation-promoting activities [13]. These physiological functions are mediated by its direct associations with tubulin/microtubules as well as with tubulin deacetylases such as histone deacetylase 6 and sirtuin-2 (SIRT2) [20,21]. The bundling and stabilization of microtubules result from the dimerization of the tubulin-attached monomeric TPPP [22]. The disordered TPPP has a zinc-finger motif and also has a Mg^2+^-dependent GTPase activity [23,24]. In exploring a potential cancer therapy, we found that TPPP has an anti-proliferative action. Indeed, injection of human recombinant TPPP into cleavage Drosophila embryos expressing a tubulin–green fluorescent protein fusion revealed that TPPP inhibits mitotic spindle assembly and nuclear envelope breakdown without affecting other cellular events [25]. This inhibition of mitosis would be consistent with the loss of TPPP allowing the proliferation of cancer cells [13] and consistent with the very low level of TPPP reported in a brain tumor (oligodendroglioma) [26]. Xie and colleagues have recently investigated the only Drosophila homolog of TPPP, the Ringmaker (Ringer) protein, and found locomotor disabilities, reduced lifespan, and neurodegeneration in adult Ringer mutants [27]. An association of Ringer with mitochondria, which resulted in ultrastructural damage and dysfunction of mitochondria coupled with increased mitochondrial superoxide levels, decreased mitochondrial membrane potential, and decreased ATP levels in the case of mutants, was also observed [27]. In the case of human TPPP, an association with mitochondria was also observed in OLGs in human control brain tissues; however, its function in mitochondria is not known [19]. PD patients often suffer from sleep disturbances, and SYN accumulation may disrupt sleep processes and the circadian rhythm. Interestingly, Barbato and co-workers have suggested that TPPP may also be a regulator of the circadian rhythm based on a TPPP knock-out mouse model [28].

Excellent, recently published reviews focus on several aspects of synucleinopathies and, in particular, on the role of SYN in these diseases [6,10,29,30]. In this article, we highlight the role of TPPP in the pathophysiology of Parkinsonism, its potential as a therapeutic target and biomarker, and possible strategies to target the TPPP and SYN hallmark proteins. 

## 2. Pathological Interaction between TPPP and SYN

An important aspect of Parkinsonism is that the partner proteins in the pathological assembly are expressed in distinct cell types in normal brain: SYN in neurons and TPPP in OLGs, respectively [17,31,32,33]. This raises the question as to which mechanism can be responsible for their co-localization.

One possibility is the cell-to-cell transmission of SYN from neurons via the extracellular space [34,35]. Exosomes, classical exocytosis, and endocytosis as well as direct penetration can be involved in the cell-to-cell transmission of SYN [36]. The levels of exosomal total and oligomeric SYN increased in plasma in PD patients as compared to healthy controls [36]. Using immunofluorescence confocal microscopy on its own or coupled with Bifunctional Fluorescence Complementation, our experiments revealed the co-location/co-enrichment of these two proteins in living human HeLa cells transiently transfected with TPPP and SYN [37]. The assembly of SYN and TPPP could be observed as well in the cells after their uptake from the medium [16,38]. This experimental setup mimics the pathological situation occurring in human brain where these proteins are transmitted via the extracellular space. The mechanisms involved in the cell-to-cell transmission of TPPP are not known. Interestingly, TPPP was detected in exosomes isolated from SH-SY5Y cells by LC-MS/MS-based label-free quantitative proteomics analysis (http://exocarta.org/gene_summary?gene_id=11076, accessed on 18 December 2023) [39]. 

In pathological conditions, another possibility of the inappropriate presence of SYN and TPPP in OLGs and neurons, respectively, might be the translation of their mRNAs. According to the data in the Human Protein Atlas, both SYN mRNA (SNCA: 243.0 nTPM and 51.5 nTPM in excitatory and inhibitory neurons, 89.6 nTPM and 181.8 nTPM in OLG precursor cells and OLGs, respectively) and TPPP mRNA (TPPP: 43.7 nTPM and 31.2 nTPM in excitatory and inhibitory neurons, 6.1 nTPM and 97.5 nTPM in OLG precursor cells and OLGs, respectively) are detected in OLGs and neurons in normal human brains (https://www.proteinatlas.org/ENSG00000145335-SNCA/single+cell+type, https://www.proteinatlas.org/ENSG00000171368-TPPP/single+cell+type, accessed on 27 October 2023). Recently, single-nucleus RNA sequencing revealed a high level of SNCA transcripts in inhibitory neurons and OLG progenitor cells but only a low level of these transcripts in excitatory neurons and mature OLGs [40]. In the case of TPPP, its mRNA is present in neurons but the protein itself is only present in OLGs [17,41]. Hence, possible pathological factors include a failure to inhibit translation of the inappropriate mRNA (an inhibition that should occur in physiological conditions) and/or a failure to degrade the newly synthesized protein; these possibilities would be consistent with the absence of the SYN protein in mature OLGs despite the presence of its mRNA. Another possibility would be a failure to put the protein in a place where it would not cause a problem.

The results of recent research are consistent with the above possibilities: the addition of human preformed SYN fibrils to mouse OLGs triggered an increase in endogenous SYN, which was critical for its aggregation with TPPP [14]; in agreement with this, in rat OLN-AS7 and human MO3.13 OLG models of MSA, TPPP transfection significantly increased the levels of both SYN mRNA and SYN protein [42]. Moreover, SYN fibrils interfered with the production of proteins associated with neuromodulation and myelination [43]. Regardless of the exact contribution of these mechanisms, SYN-TPPP assembly is characteristic of PD, DLB, and MSA and is only pathological [12].

## 3. The Role of TPPP in the Pathomechanism of Synucleinopathies: Protein Aggregation

**Figure 1:**
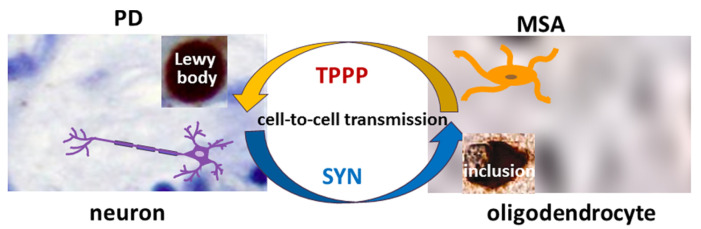
The role of SYN and TPPP in the formation of Lewy bodies and glial cytoplasmic inclusions in PD and MSA.

TPPP is co-localized with SYN in Lewy bodies in PD and DLB as well as in glial cytoplasmic inclusions in MSA [12,15] (Figure 1). Therefore, TPPP—like SYN—is considered a hallmark of synucleinopathies [12]. A TPPP-induced SYN strain had different structure and enhanced in vivo prodegenerative properties as compared to the SYN strain; moreover, injection of the preformed fibrils of the SYN-TPPP strain resulted in a shortened lifespan in a mouse model [44]. 

Most studies of TPPP have been in the context of MSA. Ota and his co-workers tested several brain tissues of MSA patients and found significantly different distributions of TPPP between the nucleus and cytosol as compared to the control [19]. The TPPP concentration increased in the cytosol at the nucleus’s expense. It is likely that this different distribution is an important factor in the progression of the disease. The distribution of TPPP inclusions within the cytosol of the OLGs of MSA patients was very different from that of the inclusions in PD, where they are concentrated in the Lewy bodies [45]. Similarly, in another study of MSA samples, TPPP was found in the enlarged oligodendroglial cytoplasm following its re-localization from the nucleus and cellular processes [46]. In MSA, extensive demyelination also occurs [18,47]. Nishimura and co-workers classified the demyelinating lesions into three stages depending on the extent of the reduction of myelin density, and SYN and TPPP-positive OLGs were frequently found in stage I but decreased in later stages [48]. The altered location and accumulation of TPPP could therefore be an early event in SYN aggregation [19,46,49]. 

Analysis of the role of TPPP also demonstrated that TPPP is enriched in SYN-bearing Lewy bodies in both PD and DLB [12,46]. The oligodendroglial pathology in DLB is generally considered less significant than in MSA. Comparison of DLB and MSA did, however, reveal both shared and distinct patterns. The immunoreactivity of TPPP in the oligodendroglia cytoplasm differed more from the controls for MSA than for DLB; a disintegration of myelin with loss of TPPP nuclear staining was characteristic of MSA, while DLB was more similar to the controls without showing a significant loss of nuclear TPPP.

## 4. The Role of TPPP in the Dysregulation of Protein Degradation in Parkinsonism

In normal human brain, levels of unwanted proteins as well as aggregates are controlled by the ubiquitin–proteasome system (UPS) and the autophagy–lysosome pathways, which can remove troublesome proteins. One target of these degradative processes is the set of intrinsically disordered proteins (IDPs) involved in the formation of toxic aggregates. Monomeric proteins such as SYN and TPPP are usually degraded by the UPS, while macroautophagy is able to degrade oligomers or aggregates ([50] and references therein). SYN can also be degraded by chaperone-mediated autophagy and endo-lysosomal degradation. Dysregulation of the cellular degradative pathways has been reported in Parkinsonism, and autophagy modulation has been suggested as a possible strategy for therapeutic intervention [51].

Our experimental studies with human living cell models, where SYN and TPPP were taken up after being added to the medium of HeLa or SH-SY5Y cells, have shown that these pathways can degrade excess or over-produced SYN and TPPP but they cannot degrade their pathological assemblies [50]. We have provided evidence that the TPPP-induced pathological SYN assemblies resist proteolytic degradation [50]. TPPP counteracted SYN degradation by hindering autophagy maturation at the stage of autophagosome formation and its fusion with lysosome [50]. This is in line with other studies, where TPPP and SYN impaired the autophagy flux in different cellular models of Parkinsonism [42,52,53]. Interestingly, the TPPP-induced inhibition of the autophagosome fusion with lysosomes also contributed to the secretion of SYN into the medium in PC12 cells with inducible expression of TPPP and SYN [53].

## 5. SYN and TPPP as Biomarkers

Synaptic dysfunction and degeneration are central contributors to the pathogenesis and progression of Parkinsonian disorders. Therefore, identification and validation of biomarkers reflecting pathological synaptic alterations are greatly needed [54]. Human cerebrospinal fluid (CSF) has a composition similar to that of the brain extracellular fluid (ECF), and the two fluids circulate freely together in the brain [55].

In a recent webinar conference organized by the Michael J. Fox Foundation entitled “Major Research Breakthrough: A New Biomarker for Parkinson’s” (20 April 2023) the significance of the SYN seed amplification assay (SAA) using CSF was highlighted [56]. This new assay may allow the aggregation of SYN to be used as a biomarker in order to distinguish people with PD from healthy controls even at an early stage of the disease. The cross-sectional analysis included 1123 participants; the assay classified PD patients with high sensitivity and specificity, and could detect low-abundancy SYN. That said, application of this assay for routine laboratory tests may prove challenging. Nevertheless, use of the SYN SAA for biochemical diagnosis of PD and MSA reveals the crucial role it could play in therapeutic development [56]. 

In the context of the exploration of candidate biomarkers for MSA, disregarding its close relationship with PD does not seem justifiable. Several recent studies have shown that SYN might be a potential diagnostic biomarker for PD in CSF though the results are inconsistent [57,58,59]. The results of a meta-analysis investigating the diagnostic and differential diagnosis efficacy of CSF SYN in PD have shown that its median concentration is significantly lower in PD compared to controls, but significantly higher in PD as compared to MSA [60,61]. However, the median concentration of CSF SYN oligomers was significantly higher in PD than in controls [60]. This could be due to the fact that the presence of TPPP in the neurons promotes the SYN assembly where these proteins are co-enriched and co-located in the well-separated, individual Lewy bodies.

**Figure 2:**
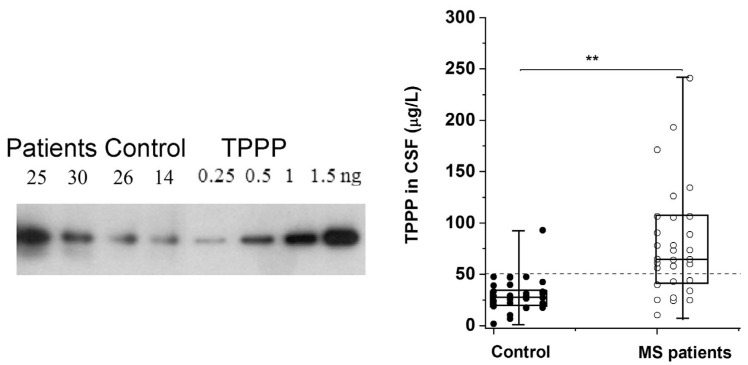
TPPP levels in the CSF of MS and non-MS patients as quantified by Western blot [63]. A representative Western blot using a specific TPPP antibody is shown for different CSF samples of patients (25 and 30, ○) and the corresponding controls (non-MS patients, ●) (14 and 26). Each box extends from the 25th to the 75th percentile with the middle line representing the median. The vertical bars indicate the full range of TPPP levels. The *p* values were determined by Mann–Whitney U tests. ** *p* < 0.000005. The dashed line corresponds to 50 μg/L [63].

The altered regulation/location of TPPP results in its pathological assembly with SYN in MSA, which leads to demyelination as in the case of Multiple Sclerosis (MS). MS is a chronic, inflammatory, demyelinating disease with a variable extent of remyelination depending on the differentiation of OLGs [62]. We have developed and validated a sensitive assay based on Western blotting coupled with chemiluminescent detection using human recombinant TPPP and CSF for the quantification of TPPP in the case of different MS patients and controls [63] (Figure 2). According to this assay, the median TPPP content of the CSF was 64.7 μg/L for patients, while it was 27.9 μg/L for non-MS patients. The higher level of TPPP in the MS patients was independent of age, gender, and the time between lumbar puncture and relapse [63]. These results suggest that (i) TPPP-based assay with CSF of the patients may be suitable for the diagnostic testing of MS patients and (ii) this assay could be used to measure TPPP levels in the CSF of MSA and PD patients. 

The novelty of our strategy for finding biomarkers for MSA and PD is the simultaneous quantification of SYN and TPPP and the calculation of the ratio of these quantities, which may allow distinctions to be made not only between patients and controls, but also between MSA and PD. These biomarkers could be used for prognosis and for monitoring the results of treatment. The fact that both SYN and TPPP are present in CSF raises the possibility of targeting them in the extracellular space. 

## 6. Targeting the Interface of the Pathological SYN-TPPP Complex

Efficient therapies and disease-modifying treatments are under intense research. A search for Parkinsonism produced 100 clinical trials, 57 of which were interventional (https://clinicaltrials.gov, accessed on 1 December 2023). Examples of these interventional trials are given in Supplementary Table S1. However, there has yet to be a clinical or preclinical trial related to TPPP as far as we are aware. 

A potential strategy to eliminate the excess of these hallmark proteins as well as their toxic aggregates includes the use of small molecules (such as Epigallocatechin gallate, Anle138b, or SynuClean-D), which have the advantages of their usually low cost, high stability, and bioavailability [64,65,66]. However, IDPs such as SYN and TPPP often lack the pocket needed to bind these molecules; an alternative strategy to inhibit protein–protein interactions is to use peptides and peptidomimetics, which is now a rapidly progressing field. Short peptides may have high specificity and low toxicity, although poor bioavailability and limited cellular uptake may pose problems [67]. Peptides that target different SYN regions have indeed been identified (Table 1) [67]. For example, Nim and co-workers found PDpep1.3 by a high-throughput, proteome-wide peptide screen; this peptide reduced the accumulation of SYN in a rat model of PD [68]. A neurohormone peptide and a cerebral dopamine neurotrophic factor (CDNF)-derived peptidomimetic also displayed beneficial effects in PD models [69,70].

A therapeutic strategy based on the targeting of either TPPP or SYN is not without risks. One reason for this is that, as IDPs, the structures of these ‘chameleon’ proteins can adopt many conformations, some of which can perturb the drug target [37,71]. As demonstrated previously, the SYN protein functions physiologically as a monomer; however, its occurrence as a metastable tetramer in a dynamic equilibrium with the monomers has also been suggested [72,73]. Another reason is that they are responsible for essential physiological functions that must be preserved. 

**Figure 3:**
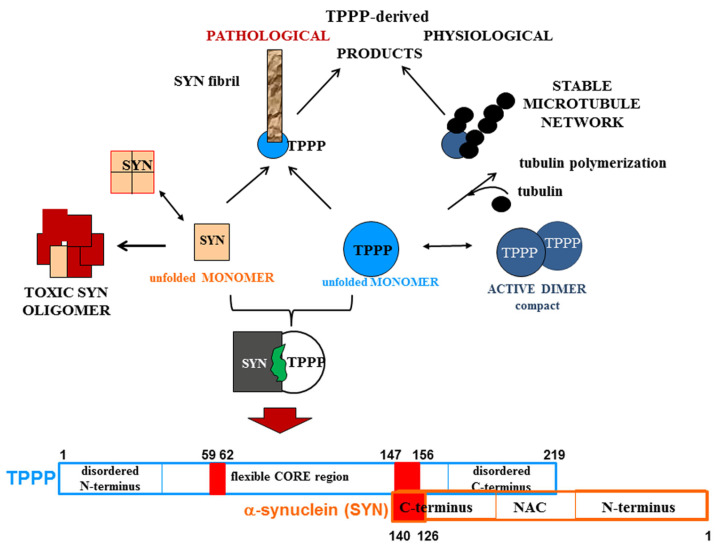
Homo- and hetero-associations of TPPP and SYN in physiological and pathological conditions and the interface segment of the SYN-TPPP complex as a potential drug target.

A strategy that we have developed and reported is the drug targeting of the hetero-complexes of SYN and TPPP [16,37,38]; this is because these complexes, which are only pathological, are the initiators of the neurodegenerative processes [12,14,74,75] (Figure 3). This strategy is based on targeting the interface where SYN and TPPP are in contact so as to trigger the disassembly of the pathological complexes [16,37,38]. The segments of the interface of both SYN and TPPP that are involved in the formation of their assemblies have been identified by studying the effects of several mutated and truncated forms of the proteins on their association [16,37,38]. Our studies revealed that the truncation of the C-terminal segment of SYN did prevent its hetero-association with TPPP [38]. This suggests that the SYN126-140 fragment may function as a competitive inhibitor of the association of the two proteins in vitro as well as in a human cell model, where the proteins were added to the medium of CHO cells [38]. Taken together, these findings substantiate drug-targeting strategies based upon (i) elimination of the toxic species; (ii) proteolytic degradation of the excess and/or pathological partner; and (iii) maintenance/recovery of the physiological function of SYN/TPPP. Moreover, strategies for selecting drugs must also take into account the physiological functions of the validated sites of the hallmark proteins. 

One of the novelties of our strategy is that it is based on the crucial role of TPPP in the formation of SYN assemblies and in the deregulation of their cellular proteolysis; hence, targeting the SYN-TPPP interaction could eliminate the toxic SYN-TPPP assemblies. For this strategy to work, we suggest that autophagy modulation by itself may not be sufficient and that the inhibition/destruction of SYN-TPPP assemblies is also necessary. Such elimination of these assemblies may require adopting and adapting new and emergent techniques. 

## 7. PROTAC and Related Technologies for the Elimination of Unwanted Proteins

**Figure 4:**
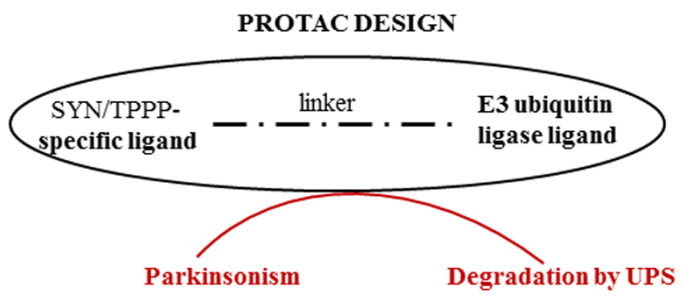
Degradation of proteins of the pathological assembly by PROTAC technology.

An effective strategy for preventing or destroying the pathological assembly of TPPP with SYN can be the PROteolysis TArgeting Chimera (PROTAC) technology [76]. This provides a way to target therapeutically attractive proteins (which are often undruggable), such as SYN or TPPP, thereby allowing the proteolytic degradation of one of the partner proteins by presenting this protein to the proteasome machinery. PROTACs may be able to overcome the inherent limitations of small-molecule inhibitors; they can be used even if there is a lack of suitable active sites to target and can reduce the risk of drug resistance [77]. In the case of synucleinopathies, this strategy is based on the degradation of inappropriate IDP and/or an excess of appropriate IDP (Figure 4). Proteolytic degradation of the accumulated SYN (PD and DLB) or TPPP (MSA) along with their pathological partner, TPPP or SYN, respectively, should then reduce the levels of these proteins and hence reduce the levels of the pathological assemblies (or eliminate them entirely). 

A chimeric compound of an E3 ubiquitin ligase ligand and a motif specific for the target protein may allow PD or MSA to be treated via the UPS. We have successfully used the PROTAC technology to study the proteolytic degradation of SIRT2 in developing a potential anti-cancer therapy based on targeting this protein [78]. Indeed, PROTAC technology has been used in clinical trials that are usually related to cancers such as NCT05654623, where a new medicine called ARV-471 (PF-07850327) is being investigated for the treatment of advanced metastatic breast cancer [79]. 

Some SYN-specific PROTAC constructs are also available [80,81,82,83,84]. These PROTAC molecules target SYN either directly or indirectly (Table 2). The choice of SYN binding motif is important since the inhibitors of SYN aggregation that are often used, such as Anle138b, can bind different SYN species. The other parts of these PROTAC constructs are usually common ligands of E3 ligases. Qu and co-workers designed an interesting chimera comprising three segments: a targeting peptide with a cell-penetrating domain, a SYN protein-binding domain, and a short, strong, proteasome-targeting motif [85]. This construct decreased the SYN level in a dose- and time-dependent manner in cultured cells and primary neurons. Lee and co-workers successfully induced the degradation of SYN aggregates by using an Autophagy-Targeting Chimera in a mouse model, without degradation of monomers [86].

The UPS degrades monomeric SYN and partially small, soluble oligomers, but cannot degrade larger SYN species, especially aggregates. However, autophagy is often compromised in Parkinsonian patients. These factors should be taken into account in designing and optimizing effective chimeras. No clinical trial for the treatment of synucleinopathies was found when we searched the https://clinicaltrials.gov site (accessed on 1 December 2023). Nevertheless, PROTAC and related technologies remain a promising way to obtain anti-Parkinsonism agents. The use of these technologies by exploiting specific discovered and new ligands of SYN and/or TPPP could constitute a credible therapeutic strategy. 

## 8. Targeting mRNA in Parkinsonism

One possible approach is to inhibit gene expression and reduce protein levels by the use of small interfering RNA (siRNA), microRNA (miRNA), or short hairpin RNA (shRNA). The advantages of these molecules include that they are cost effective, are relatively simple and rapid to produce, can be designed in many different ways, and can target previously undruggable targets [87,88]. However, the lack of specificity of miRNAs can be a serious drawback since they are not specific for single mRNAs. miRNAs or siRNAs used for intervention are usually limited to clinical trials related to cancer such as NCT01591356 or NCT03608631 [89]. Clinical trials investigating the relevance of miRNAs or siRNAs to Parkinsonism can be found but, in these trials, miRNAs are used as biomarkers and not as therapies (such as NCT03466723 and NCT02672943) (https://clinicaltrials.gov, accessed on 1 December 2023). However, antisense oligonucleotides (ASOs) can decrease the SYN level by targeting its RNA intracellularly [90], and there is one ongoing trial (NCT04165486) where such a compound (ION464) is being tested on MSA patients.

miRNAs are now known to be key regulators of gene expression leading to neurodegenerative disorders [91]. Recently, Noronha and co-workers carried out a meta-analysis on differentially expressed miRNAs in neurodegenerative diseases [92]. Their analysis included ten studies on PD and seven on MSA but only one on DLB. They found that the number of differentially downregulated miRNAs was higher than the upregulated ones. However, the overlap of these miRNAs between PD and MSA was below 10%. Twenty-two miRNAs were downregulated which were common to MSA and PD. In the case of PD, some naturally occurring miRNAs have been discovered that directly or indirectly impact SYN expression/accumulation as well as the mechanism of action such as miR-7 and miR-153 (Table 3) [93,94,95]. The role of these miRNAs in downregulating SYN has been shown both in vitro and in animal experiments [96]. miR-7 directly downregulates SYN level, since it binds to SNCA mRNA 3′-UTR, although it also targets other mRNAs involved in neuronal homeostasis [97,98]. 

In the case of synucleinopathies, the mutual presence of the two hallmark proteins is crucial for the maturation of the toxic assemblies since their hetero-association leads to PD or MSA. Consequently, a reduction in the levels of these proteins by specific miRNAs should also result in a reduction in the hetero-associations. The feasibility of the RNA strategy for arresting the differentiation of OLGs was demonstrated in our lab in a collaboration with Pierre Lau of NIH: this entailed the downregulation of TPPP in living progenitor OLG (CG-4) cells using a specific miRNA (miRNA-206) [17]. This inhibition of TPPP by specific oligonucleotides revealed the crucial physiological role of TPPP in the differentiation of the progenitor OLGs. miRNA-206 also plays an important role in the pathomechanism of various diseases [103,104]. It was suggested as a possible biomarker and a therapeutic target in Alzheimer’s disease. The concentrations of beta-amyloid, tau, and miRNA-206 were intended to be studied in a clinical trial (NCT02129452), but no result has been reported as yet. microRNA-1 has also been found to downregulate TPPP in the hippocampus [105].

siRNAs are small interfering RNAs that are artificially synthesized as 19–23 nucleotide-long, double-stranded RNA molecules. We have reported that selected siRNAs can also reduce TPPP levels and inhibit the differentiation of OLGs [17]. Therefore, the downregulating effects of siRNA and miRNA make them promising candidates for reducing TPPP levels as part of an RNA-based, therapeutic strategy for MSA similar to that proposed for PD [96]. Nevertheless, more work is needed if we are to better understand the roles of miRNAs in neurodegeneration.

## 9. Conclusions and Beyond

New ideas and new findings in the field of neurodegenerative diseases hold the promise of new diagnostic techniques and therapies. Focusing on synucleinopathies, these ideas include the Sherpa hypothesis which proposes that Phenotype-Preserving Disordered Proteins (PPDPs), such as TPPP and SYN, and oligomeric structures as well as the homo- and hetero-associations of these disordered proteins can generate distinct, physiologically/pathologically appropriate phenotypes [106]. SYN and TPPP as PPDPs assist in the maintenance of the phenotypes of neurons and OLGs despite these cells being subject to stresses that might otherwise disrupt their phenotypes [106]. The dynamic microtubule system makes a powerful contribution to the generation of coherent phenotypes by binding certain proteins/enzymes such as SYN and TPPP [106,107]. The disturbance of the multiple functions of the microtubule network is particularly important in brain cells, as observed in a variety of neurodegenerative disorders [108,109]. The Sherpa hypothesis is supported by the finding that the physical interaction between TPPP and SYN (which are not normally found together) leads to inappropriate phenotypes with pathological consequences such as those due to abnormal proteolytic degradation. This pathological interaction between the two PPDPs has implications for the identification of new biomarkers for neurodegenerative diseases where, in the case of MSA and PD, we propose using both the SYN and TPPP levels and/or the ratio of SYN to TPPP in the CSF. This pathological interaction also has implications for therapy. We therefore advocate strategies that degrade the inappropriate PPDP (e.g., via the use of SYN-specific or TPPP-specific PROTAC constructs), that reduce the levels of the PPDPs (e.g., via siRNA and miRNA, autophagy activation), and that prevent interaction between the PPDPs by interface targeting (via the use of competitive inhibitors such as peptide fragments).

Devising new therapies for Parkinsonism would be greatly facilitated by a better understanding of the physiological and pathological mechanisms responsible for this and related diseases. The pathophysiology of the assembly/aggregation of SYN resulting from interaction with TPPP is a complex process that is yet to be understood in detail; this incomplete understanding comes from the fact that the two proteins are significantly co-enriched and co-located in inclusions in neurons (PD and DLB) and OLGs (MSA) whilst still being expressed endogenously in two different cell types of the normal human brain [17,31,32,33]. One of the limitations of Parkinsonism research is the neglect of the key role of TPPP; this neglect may be due to the present lack of knowledge of the molecular mechanism of cell-to-cell transmission of SYN (neuron to OLG) and TPPP (OLG to neuron). How serious is this lack of knowledge? Could remedying it make a major contribution to the discovery of specific drugs for PD and MSA patients? Re-localization of TPPP in the OLGs is an early event in the pathomechanism of MSA, which precedes the formation of inclusion bodies [19,46,49]. This fact again emphasizes the importance of TPPP as a drug target. Another important question is whether the inappropriate presence of TPPP is an early or late event in the pathology of PD. A TPPP-knockout rodent model provided useful insights into the physiological function of TPPP [110] but, from the point of view of Parkinsonism, a TPPP-overexpressing rodent model may contribute to a better understanding of the underlying pathological processes. Both in vivo models that include TPPP and observational studies in patients are needed to clarify these questions. 

Better treatments/therapies for PD and MSA could clearly result from a better understanding of the pathophysiology of these diseases, which may entail introducing new concepts. In summary, developing successful therapies requires the identification and validation of the distinct pathomechanisms of neurodegenerative diseases, taking into account the potency of TPPP as a pathological partner. 

## Figures and Tables

**Table 1:** Examples of peptides/peptidomimetics as possible anti-Parkinsonism agents.

Peptide	Target	Effect	Reference
PDpep1.3	SYN	reduced SYN accumulation in *C. elegans* and rat models	[68]
Kisspeptin-10 ^1^	SYN	mitigated SYN-induced toxicity in SH-SY5Y cells	[69]
HER-096 ^2^		reduced SYN aggregation and modulated the unfolded protein response pathway in vitro and in a mouse model	[70]

^1^ Kisspeptin-10 is a hypothalamic neurohormone-derived peptide. ^2^ HER-096 is a cerebral dopamine neurotrophic factor (CDNF)-derived brain-penetrating peptidomimetic.

**Table 2:** PROTAC constructs for Parkinsonism.

Compound	Design	Effects	Reference
Anle138b-centered PROTACs	Anle138b is a SYN binder and aggregation inhibitor; lenalidomide and thalidomide as E3 ligase Cereblon ligand	decreased SYN aggregation in neuronal cells	[82]
sery384-based PROTACS	sery 384 is a SYN aggregation inhibitor; common ligands of E3 ligases (Cereblon, von Hippel–Lindau, cIAP1)	induced degradation of SYN aggregates in a dose- and time-dependent manner in cells	[83,84]
PD163916	an established active-site inhibitor of p38 MAPK, resulting in proteasome activation	decreased SYN levels in cells	[80]
peptide TAT-PBD-PTM	targeting peptide with a cell-penetrating domain (TAT), a SYN protein-binding domain (PBD), and a short strong proteasome-targeting motif (PTM)	decreased the SYN level through proteasome degradation in a dose- and time-dependent manner in cultured cells and primary neurons	[85]
ATC161, a Autophagy-Targeting Chimera	bind both SYN aggregates (via Anle138b) and p62/SQSTM1/Sequestosome-1, an autophagic receptor	induced selective degradation of SYN aggregates but not monomers in cells and a mouse model; improved muscle strength and locomotive activity in mouse	[86]

**Table 3:** Examples of interventions targeting mRNA in Parkinsonism.

Compound	Target	Effects	Reference
Synucleozid	SNCA mRNA 5′ UTR at the iron-responsive element	decreased SYN level	[99]
quercetin	pri-miR-7/HuR interaction inhibitor	upregulated miR-7, decreased SYN level	[100]
miR-7	3′-untranslated region of SNCA mRNA	reduced SYN levels, protected neuronal cells, and protected against preformed fibrils in mice	[97]
GRK2-specific miRNA	G-protein-coupled receptor kinase 2 (GRK2)	reduction in high-molecular-weight SYN species and neuroprotection in a mouse model	[101]
shRNA-minicircles delivered by RVG-extracellular vesicles		SYN downregulation in mice	[102]
miRNA-206	TPPP	downregulation of TPPP in progenitor OLGs ^1^	[17]

^1^ This was not a cell model of Parkinsonism.

## Data Availability

No new data were created or analyzed in this study. Data sharing is not applicable to this article.

## Supplementary Materials

The following supporting information can be downloaded at https://www.mdpi.com/article/10.3390/cells13040338/s1, Table S1: Examples of interventional clinical trials in Parkinsonism.
